# Can tissue immune states shape the risk of hyperprogressive disease?

**DOI:** 10.3389/fimmu.2026.1823041

**Published:** 2026-08-11

**Authors:** Iván M. Moya

**Affiliations:** Cancer Research Group, School of Biotechnology, Faculty of Engineering and Applied Sciences, Universidad de Las Américas, Quito, Ecuador

**Keywords:** cancer, hyperprogressive disease, immune checkpoint inhibitors, PD-1, tumor immune microenvironment

## Abstract

Immune checkpoint inhibitors (ICIs) have transformed cancer therapy by unlocking antitumor T-cell responses across multiple cancer types. However, a subset of patients experience rapid acceleration of disease progression shortly after ICI initiation, a phenomenon termed hyperprogressive disease (HPD). Although HPD remains clinically heterogeneous, emerging evidence suggests that HPD is not solely a stochastic anomaly, but may result from an immune rewiring failure triggered by checkpoint release in chronically inflamed, myeloid-dominated, or tolerogenic host organs that are permissive for tumor growth. Mechanistic studies in metabolic dysfunction-associated steatohepatitis (MASH)-associated hepatocellular carcinoma show that PD-1 blockade can fail to restore effective antitumor immunity and instead exacerbate pathogenic T-cell-mediated injury. Related findings in colitis-associated colorectal cancer, chronic lung inflammation, and glioblastoma suggest how pre-existing immune circuits shaped by inflammation, fibrosis, metabolic dysfunction, or immune privilege can constrain productive immune rewiring. We synthesize these organ-specific archetypes into an organ-context model in which ICIs act not by generating *de novo* immunity, but by amplifying pre-existing immune programs. In this model, checkpoint release may lead to either productive tumor control or maladaptive activation and accelerated progression, depending on the baseline immune architecture of the host tissue. Understanding HPD through the lens of organ-context immune wiring has major translational implications for predictive biomarkers, stratification strategies, and the design of context-aware immunotherapy.

## Hyperprogressive disease

Immune checkpoint inhibitors aim to restore T-cell-mediated tumor recognition and cytotoxic control across multiple cancer types (1). However, the therapeutic response remains heterogeneous (1), and in a subset of patients, disease progression appears accelerated following initiation of immunotherapy (2, 3). This clinical phenomenon, known as hyperprogressive disease (HPD), has been reported across multiple tumor types, with estimated incidence ranging from approximately 4% to nearly 30%, depending on tumor context and diagnostic criteria (2–6). This variability has made HPD both clinically important and biologically difficult to interpret, and has contributed to its perception as a paradoxical response pattern.

A central challenge in diagnosing HPD is that it remains clinically heterogeneous and poorly defined. Across studies, HPD has been defined using different approaches, including acceleration of tumor growth rate, changes in tumor growth kinetics, RECIST-based progression, early time-to-treatment failure, or composite criteria (2–9). These definitions do not always identify identical patient populations, and many retrospective studies lack robust pretreatment growth kinetics. As a result, the field continues to debate whether HPD represents a distinct treatment-associated biological phenomenon, the natural history of highly aggressive tumors, or a mixture of both. In this review, we use HPD as a clinical pattern of unusually rapid post-ICI progression and focus on mechanisms that may render this pattern more likely in specific tumor and tissue contexts, rather than assuming that all cases reflect a single biological process.

Despite these diagnostic uncertainties, mechanistic investigations have begun to identify tumor-intrinsic, stromal, and immune processes that may contribute to some HPD-like outcomes (10–16). Among the identified mechanisms, HPD has been associated with amplification of the *MDM2* and *MDM4* loci, encoding mouse double minute 2 homolog and mouse double minute 4 homolog, respectively, and alterations in epidermal growth factor receptor (*EGFR*) signaling, as well as immune-modulating factors such as proliferation of regulatory T-cells following PD-1 blockade (10, 13–16). Additional mechanisms, including baseline tumor growth kinetics, tumor burden, stromal organization and treatment-specific factors, may also contribute to early acceleration after immunotherapy (3, 11, 13, 15–17). Yet, although these features are associated with poor outcomes and early treatment failure, they do not fully explain the pronounced variability in HPD observed across tumor types and anatomical sites (13, 17).

**Figure 1:**
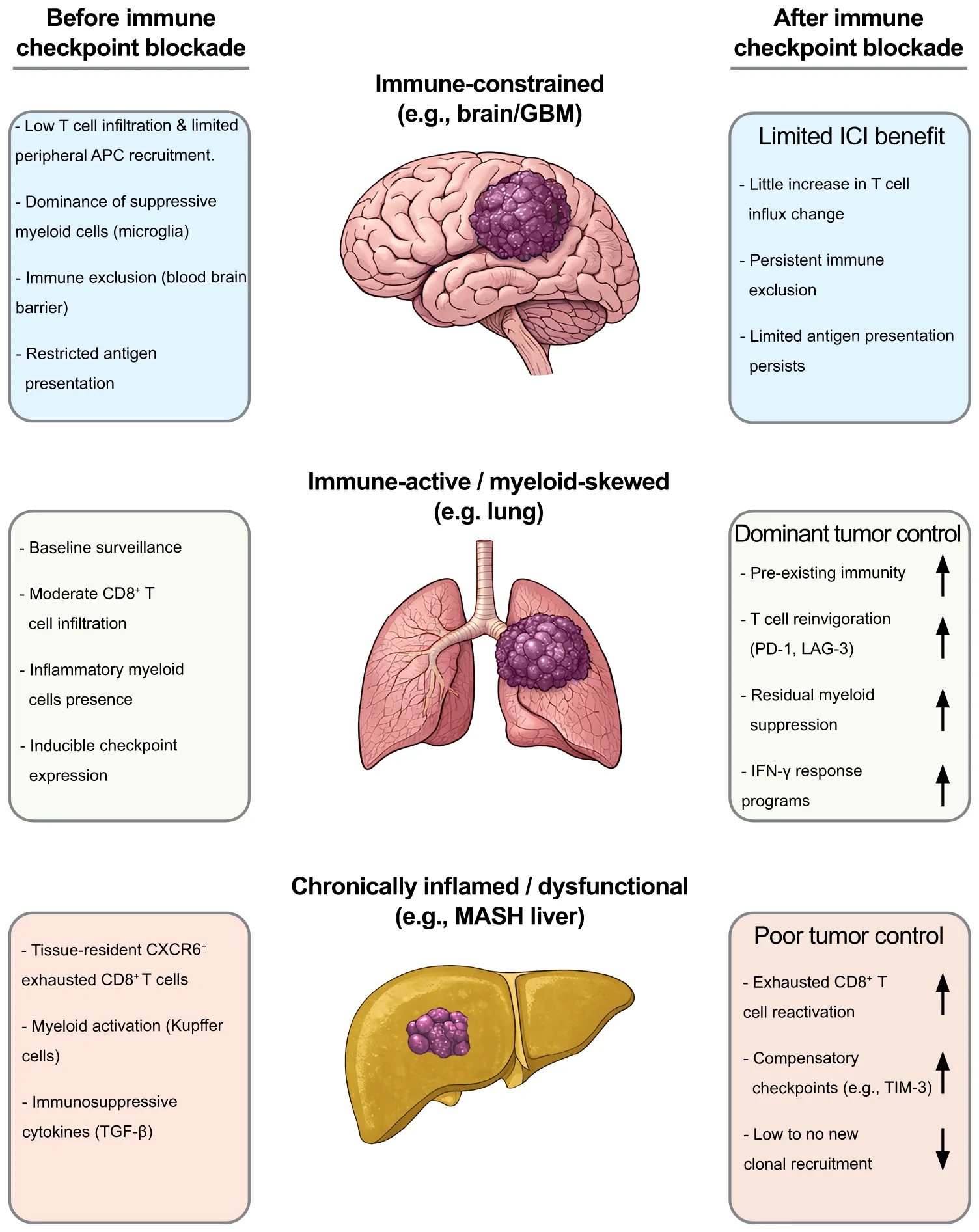
Baseline tissue immune states and post-ICI immune rewiring. Schematic comparison of three representative tissue immune contexts before and after immune checkpoint blockade. The immune-reactive lung context is shown with pre-existing immune surveillance, CD8^+^ T-cell infiltration, inflammatory myeloid cells, and inducible checkpoint expression. The chronically inflamed MASH liver context is shown with tissue-resident dysfunctional CD8^+^ T-cell states, Kupffer-cell or myeloid activation, and immunosuppressive cytokine signaling. The immune-constrained brain context is shown with restricted immune access, low T-cell infiltration, suppressive myeloid dominance, and limited antigen presentation. These baseline states can bias post-ICI rewiring toward tumor control, resistance, or maladaptive acceleration.

An emerging body of translational and preclinical work indicates that the immune landscape of the host organ, including chronic inflammation and regulatory circuitry, can shape the outcome of immune checkpoint inhibition (12, 18–20). In particular, organs subject to chronic inflammation or immune privilege can sustain cellular programs that limit productive immune rewiring following checkpoint inhibition (21). Preclinical studies in MASH-associated hepatocellular carcinoma show that PD-1 blockade can fail to restore effective antitumor immunity and, in some models, exacerbate pathogenic T-cell-mediated tissue damage (12). Similar patterns have been reported in inflammation-associated colorectal cancer, where PD-1 blockade increased tumor growth in pre-existing inflammatory settings (20). Together, these observations place organ immune state alongside tumor-intrinsic, stromal, systemic, and treatment-related factors as a variable that can influence post-ICI tumor trajectories (**Figure 1**).

**Figure 2:**
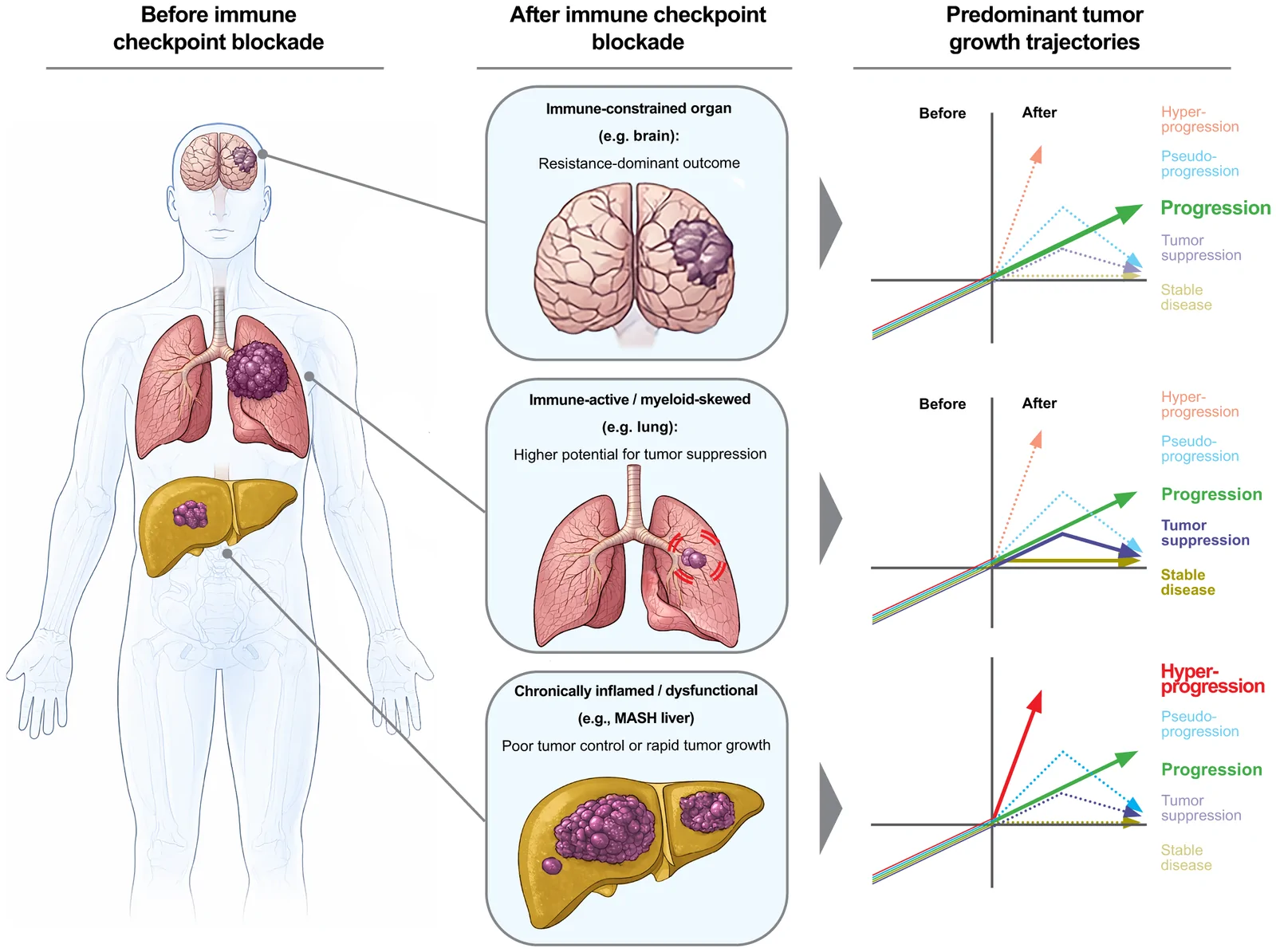
Tissue immune states and post-ICI tumor growth trajectories. Schematic representation of how selected tissue immune contexts can bias post-ICI tumor growth trajectories. The immune-constrained brain/GBM context is shown as resistance-dominant, the immune-active/myeloid-skewed lung context is shown as heterogeneous and potentially responsive, and the chronically inflamed MASH liver context is shown as vulnerable to poor tumor control or HPD-like acceleration in selected settings. Repeated trajectory labels are shown for each context to indicate the full range of possible outcomes. Line thickness and label emphasis indicate the trajectory highlighted in the schematic, whereas faded labels and thinner lines indicate alternative documented outcomes. The curves represent relative changes in tumor burden over time, not absolute probabilities.

Here, we examine HPD as a context-dependent clinical pattern that may arise more readily when checkpoint inhibition occurs in tissue environments shaped by chronic inflammation, myeloid regulation, immune tolerance, or restricted immune access (1, 12, 22). Thus, while ICI therapy can result in tumor control, under certain immunological constraints it may induce pathogenic activation and accelerated disease progression (11, 12, 20). Therefore, we ask how baseline organ immune states influence post-checkpoint immune rewiring and how these tissue conditions may bias tumor growth trajectories after therapy. The sections below first summarize baseline tissue immune states and immune rewiring after checkpoint inhibition, then organize post-ICI clinical trajectories, and finally compare these principles across liver, colon, lung, and brain (**Figures 1**, **2**).

## Checkpoint inhibition is shaped by pre-existing tissue immune states

Immune checkpoint inhibitors (ICIs), rather than generating *de novo* immune responses, seem to act by lifting inhibitory signals that constrain pre-existing immune programs (1, 22). In tumors characterized by T-cell-inflamed microenvironments, such as melanoma, checkpoint blockade can enable reinvigoration of CD8^+^ T-cell function and lead to tumor suppression in a subset of patients (22–24). However, in tissues with pre-existing immune dysregulation, checkpoint blockade can fail to restore effective cytotoxicity and, in certain contexts, may be associated with pathogenic immune activation or tumor growth acceleration (11, 12, 20). These divergent outcomes are shaped in part by the immune architecture of the tumor-bearing tissue, together with tumor-intrinsic, stromal, treatment-related, and systemic host factors. We propose a conceptual model in which baseline immune state, patterns of immune rewiring, and resultant outcome trajectories are considered together to explain how checkpoint blockade can lead to tumor control, resistance, or maladaptive progression.

## Baseline organ immune state

Tissues differ markedly in their steady-state immune tone, shaped by developmental programs, barrier functions, microbial exposure, and cumulative inflammatory history (**Figure 1**) (25–27). This intrinsic heterogeneity can shape how checkpoint blockade is interpreted by tissue-resident immune circuits and thus influence therapeutic outcomes (28–30). Checkpoint blockade interacts with organ-specific immune architectures that can condition the quality and trajectory of the immune response (12, 20, 26). In immunologically quiescent or surveillance-prone tissues such as the skin, tumors often arise within a microenvironment enriched for tissue-resident memory T-cells and antigen-presenting cells (31). In melanoma and related cutaneous malignancies, this baseline immune readiness is associated with effective reinvigoration of cytotoxic T-cells following checkpoint blockade, leading to durable tumor control in a subset of patients (22, 24). Thus, inhibitory checkpoint pathways may restrain pre-existing antitumor immunity, and their blockade can restore productive cytotoxic responses when tumor immune programs remain functionally recoverable.

By contrast, tumors developing in chronically inflamed organs encounter a different immune landscape (12, 18, 29). The liver in MASH, the colon in inflammatory bowel disease, and the lung in chronic inflammatory conditions such as COPD can harbor immune circuits shaped by persistent antigen exposure, myeloid skewing, cytokine imbalance, and tissue remodeling (12, 20, 32). These environments can harbor exhausted T-cell populations, promote expansion of suppressive myeloid cells, and develop fibrosis-associated barriers that hamper immune cell function (12, 18, 32). Although chronic inflammation alone is unlikely to determine therapeutic response or HPD risk, exhausted T-cell states may be epigenetically stabilized in chronic inflammation, which can limit the capacity of checkpoint blockade to restore cytotoxic activity (12, 30, 33). Instead, release of inhibitory signals can exacerbate dysfunctional immune programs, potentially predisposing to maladaptive responses following therapy. Thus, the composition, severity, spatial organization and cellular balance of the inflammatory state are likely to influence whether checkpoint release promotes tumor control, resistance, toxicity, or maladaptive progression. This distinction is particularly relevant in settings such as COPD-associated lung cancer, where clinical associations with ICI outcome remain heterogeneous.

A distinct but related challenge is posed by immune-constrained or tolerogenic organs, including the brain and, under physiological conditions, the liver, which exhibits features of immune tolerance (34, 35). These tissues actively suppress inflammation to preserve organ integrity, employing both structural and molecular mechanisms that limit immune infiltration and activation (36, 37). In glioblastoma, for example, profound baseline T-cell dysfunction and restricted immune access can constrain the efficacy of immune checkpoint inhibitors, while PD-1 blockade has shown limited clinical benefit in many patients with recurrent disease (30, 38). In these settings, checkpoint inhibition may be insufficient to overcome dominant tolerance programs, even in the presence of tumor antigens. Importantly, these organ states are not mutually exclusive as a single tumor-bearing organ can combine surveillance-prone, chronically inflamed, fibrotic, myeloid-rich, or tolerogenic features. Thus, the balance among these features is likely to shape the overall response to checkpoint blockade.

## Immune rewiring following checkpoint blockade

Checkpoint inhibitors do not act on isolated T-cells but perturb immune circuits already in motion (39). Their effects therefore depend on how the broader immune ecosystem, including stromal, regulatory T-cell, and myeloid elements, responds once inhibitory signaling is relieved (40). In tissues with pre-existing immune dysregulation, this rewiring can follow non-productive or maladaptive trajectories (12, 20, 41). A recurring feature of chronically inflamed tumors is enrichment of suppressive myeloid populations, including myeloid-derived suppressor cells (MDSCs) and tumor-associated macrophages (TAMs) (29, 32). In the lung and liver, these cells can regulate local immune responses through arginase activity, production of IL-10 and TGF-β, generation of reactive oxygen species, and modulation of antigen presentation (29, 32, 42). Checkpoint blockade may fail to override these suppressive networks, allowing myeloid-driven regulation to persist despite T-cell–intrinsic activation signals.

Cytokine feedback loops further shape post-checkpoint immune rewiring (43). Inflammatory cytokines such as IL-6, IL-10, IFN-γ, and TGF-β may be amplified following checkpoint inhibition, particularly in chronically inflamed tissues (44, 45). These cytokines can reinforce T-cell exhaustion, sustain suppressive myeloid populations, or drive tissue damage, thereby amplifying immune dysfunction rather than productive antitumor immunity (44). In parallel, compensatory upregulation of alternative inhibitory receptors can limit the durability of checkpoint-induced activation (46). Exhausted or maladaptively activated T-cells can increase expression of TIM-3, TIGIT, LAG-3, and VISTA following PD-1 or CTLA-4 blockade, thereby maintaining inhibitory pressure through parallel immunoregulatory pathways (47). Such compensatory or refractory states are best established in selected tumor and chronic-inflammation settings and are particularly prominent in glioblastoma, where PD-1-positive CD8 T-cells can exhibit severe exhaustion signatures and limited reinvigoration capacity (30). Thus, post-checkpoint immune rewiring reflects the interaction between pre-existing tissue immune state, tumor-intrinsic features, stromal context, myeloid regulation, and compensatory regulatory circuits, rather than a uniform consequence of checkpoint release.

## Outcome trajectories: response versus dysregulation

Post-ICI outcomes can be organized as a spectrum of clinical trajectories rather than as a binary distinction between response and progression (**Figure 2**) (22, 45). In tumors with recoverable antitumor immunity, checkpoint inhibition can reinvigorate cytotoxic T-cell activity, support antigen presentation, and promote tumor regression (22, 24, 45, 48). These positive responses are often associated with transient IFN-γ signaling and clonal expansion of tumor-reactive CD8 T-cells (22, 24, 45). In contrast, in chronically inflamed, myeloid-rich, tolerogenic, or immune-constrained tissues, checkpoint release may fail to restore effective cytotoxicity and can instead coincide with immune dysfunction, tissue-damaging inflammation, primary resistance, or accelerated tumor growth (12, 20, 49).

Four clinically relevant post-ICI tumor trajectories can be distinguished. Productive response includes tumor regression or durable disease control and is usually associated with recoverable antitumor immunity. Primary progression reflects continued tumor growth without evident clinical benefit. Pseudoprogression refers to transient radiological enlargement or the appearance of new lesions followed by subsequent tumor suppression. HPD represents a more severe pattern, characterized by unusually rapid tumor growth acceleration shortly after ICI initiation (2–4, 6, 15). Unlike pseudoprogression, HPD is associated with poor clinical outcome and rapid clinical deterioration (2–4, 6). However, HPD has been difficult to distinguish consistently from aggressive natural history because published studies use different diagnostic criteria, including tumor growth rate, tumor growth kinetics, RECIST-based progression, early time-to-treatment failure, or composite definitions (6–9, 13, 15). Thus, post-ICI outcome constitutes a spectrum of trajectories shaped by tumor genotype, antigenicity, treatment regimen, stromal structure, systemic host factors, and tissue immune state.

This trajectory-based classification links immune rewiring to organ-specific evidence. The following section examines how selected organs differ in recoverable antitumor immunity, chronic inflammation, myeloid regulation, and immune access, and how these differences may bias post-ICI outcomes.

## Organ-specific archetypes: liver, colon, lung, brain

Checkpoint inhibitors act within immunological ecosystems that differ across tissues (28). These ecosystems are not neutral: they influence the direction of immune rewiring following checkpoint inhibition (41). The organ examples below compare how tissue immune states shape checkpoint responses across inflammatory, myeloid-rich, and immune-constrained settings. The liver provides the strongest experimental evidence for maladaptive post-ICI immune rewiring in a chronically inflamed organ, whereas the colon, lung, and brain provide additional but more heterogeneous evidence, including inflammation-associated tumor promotion, context-dependent ICI responsiveness, and immune-constrained checkpoint resistance.

## Liver: the paradigm case of pathogenic rewiring

The liver provides one of the clearest experimental examples of how chronic inflammation can create an immune landscape that is poorly suited to productive checkpoint reinvigoration and, in selected settings, even permissive to maladaptive immune activation upon checkpoint release (12). Immune checkpoint therapy in hepatocellular carcinoma (HCC) arising from livers with MASH not only failed to control tumor growth but exacerbated tumor burden (12). Mechanistically, CD8^+^ T-cells in MASH livers exhibited a pathogenic activation state, producing inflammatory mediators and tissue-damaging cytokines, which, when unrestrained by PD-1, amplified liver injury and tumor progression. This study showed that checkpoint inhibitors, when deployed in the wrong immune context, such as in chronically inflamed livers, can convert CD8^+^ T-cells from tumor-suppressive to tumor-promoting agents.

Critically, this phenomenon seemed to be context-specific, since the same tumor model in a non-inflamed hepatic environment responded differently (12). This supports a model in which the host organ immune state, rather than tumor-intrinsic genetics alone, can influence the polarity of checkpoint response. This concept is further supported by studies in non-MASH liver models (50). In early-stage HCC without fibrosis, metabolic dysregulation such as oxidized low-density lipoprotein accumulation drives CD36-dependent iron overload in CD8^+^ T-cells, resulting in ferroptosis-like stress and functional exhaustion (50). In this model, inhibition of CD36 or activation of the antioxidant regulator NRF2 mitigated lipid peroxidation, restored CD8^+^ T-cell effector function, and improved antitumor responses, indicating that metabolic inflammation can undermine checkpoint efficacy even outside overt MASH contexts. Thus, although not all steatotic or inflamed HCC will undergo HPD after checkpoint blockade, these results show that metabolic liver immune states can impair antitumor immunity and checkpoint responsiveness (12, 50–52). Therefore, hepatic immune context modifies but does not determine therapeutic outcome.

## Colon: colitis-associated cancer and immune misfiring

The colon, especially under conditions of chronic inflammation, provides an emerging example of maladaptive immune rewiring (53, 54). Inflammatory bowel disease (IBD) and colitis-associated cancer (CAC) arise within chronic inflammatory milieus defined by persistent cytokine signaling, including IL-6 and IL-17, epithelial barrier disruption, microbial stimulation, and immune dysfunction that can drive T-cell attenuation and altered stromal remodeling (55, 56). In preclinical models of CAC, tumor-infiltrating CD8^+^ T-cells can acquire exhaustion phenotypes marked by high expression of PD-1 and other inhibitory receptors (TIGIT, LAG-3, CD160), and transcriptional regulators such as TOX are implicated in maintaining dysfunctional states (20, 57). Recent work further indicates that CD160-expressing CD8^+^ T-cell states can influence anti-PD-1 responsiveness in MSI-H and inflammation-induced CRC models, highlighting that exhausted CD8^+^ T-cell programs are functionally heterogeneous rather than uniformly refractory to checkpoint blockade (58). Such dysfunctional states can be reinforced by transcriptional and epigenetic programs that limit productive reinvigoration after PD-1 blockade. In these preclinical inflammatory settings, PD-1 blockade has been reported to increase tumor burden without aggravating histological colitis, with a context-dependent tumor-promoting effect in inflammatory CAC models (20, 57). Clinically, this inflammatory archetype must be distinguished from dMMR/MSI-H colorectal cancer, where high neoantigen load and T-cell infiltration are associated with checkpoint responsiveness, and from pMMR/MSS colorectal cancer, which is more often immune-excluded and resistant to single-agent checkpoint blockade (59–61). Thus, the colon supports the broader organ-context model, but the direct mechanistic evidence linking colitis-associated cancer to HPD-like acceleration after checkpoint blockade remains less extensive than the evidence available in steatohepatitis-associated HCC.

## Lung: chronic inflammation and myeloid dominance

Chronic inflammatory insults in the lung, such as those associated with tobacco smoke and chronic obstructive pulmonary disease (COPD), can reshape the tumor immune microenvironment and alter responses to immune checkpoint blockade (32, 62, 63). In non-small-cell lung cancer (NSCLC), the immune landscape can include abundant neutrophil and myeloid populations, with neutrophils as the most abundant immune cell type in tumor samples and associated with immunosuppressive functions that may impede effective cytotoxic T-cell responses (32). COPD can alter immune cell composition in NSCLC, including shifts in Th1/Th17 balance and checkpoint expression, and is associated with changes in immunotherapy outcomes in clinical cohorts (62, 63). Recent meta-analytic data indicate that comorbid COPD can be associated with improved overall and progression-free survival after ICI therapy, rather than uniformly impaired outcome (64). In advanced NSCLC, favorable ICI efficacy was reported in patients with pre-existing COPD or CT-defined emphysema, including improved response and survival endpoints (65). However, COPD severity may matter, as milder COPD is associated with more favorable outcomes whereas severe COPD appears less consistently beneficial in anti-PD-(L)1-treated NSCLC (66). Thus, mild or moderate COPD and emphysema may mark a lung immune state with pre-existing immune activation, smoking-associated antigenicity, and checkpoint-pathway engagement that can favor ICI responsiveness, whereas more advanced COPD may combine immune dysregulation with reduced physiological reserve and greater pulmonary toxicity risk. In contrast to MASH-associated HCC, where metabolic inflammation can amplify pathogenic CD8^+^ T-cell programs, the lung shows that tissue inflammation can bias checkpoint responses in different directions depending on immune composition, antigenicity, organ physiology, and disease severity.

## Brain: immune privilege and inaccessible exhaustion

Glioblastoma (GBM) represents an immune-constrained checkpoint resistance state rather than a classical HPD setting, in which restricted immune access, entrenched T-cell dysfunction, myeloid suppression, treatment timing, corticosteroid exposure, and baseline tumor immune state may jointly limit effective checkpoint responses (67–69). The central nervous system limits and compartmentalizes immune cell entry under homeostasis, and many of these barriers persist in the GBM microenvironment, which is often poorly infiltrated by cytotoxic lymphocytes and enriched for myeloid suppressors and regulatory networks (69). In GBM, tumor-infiltrating lymphocytes (TILs) exhibit some of the most pronounced T-cell exhaustion signatures among human cancers, with high expression of inhibitory receptors such as PD-1, TIM-3, and LAG-3 and limited signs of functional reinvigoration in *ex vivo* assays (30). In this setting, limited ICI activity appears less consistent with hyperactivation of effector immunity than with entrenched T-cell dysfunction and multiple barriers to effective immune access. Consistent with this, single-agent PD-1 blockade has shown limited clinical benefit in most unselected or recurrent GBM cohorts (38). However, a recent neoadjuvant triplet checkpoint blockade study in newly diagnosed GBM induced marked TIL infiltration and activation after treatment, indicating that immune access and activation are not categorically impossible in GBM (70). Together, these findings place GBM as a resistance-dominant context in which immune access, T-cell state, myeloid suppression, corticosteroid exposure, treatment timing, and combination strategy may influence whether checkpoint blockade fails or induces productive immune activation (38, 70).

## Conclusions and future directions

The cumulative evidence reviewed here suggests that immune checkpoint inhibitors do not operate in an immunological vacuum (1, 28). Instead, they act upon a pre-configured immune terrain that is deeply shaped by the tissue of origin, local inflammatory history, and structural immunology of the host organ (26, 28). As such, hyperprogressive disease may not be a purely unpredictable anomaly, but may represent, in selected pathological scenarios, a context-dependent but likely predictable trajectory when checkpoint inhibition is introduced into chronically dysregulated, myeloid-rich, or immune-constrained environments (12, 30, 49). This reconceptualization has significant implications across four domains:

### Mechanistic interpretation of HPD

Rather than focusing solely on tumor-intrinsic mutations (such as MDM2 amplification or EGFR alterations), future research should give greater weight to organ-specific immune wiring (12, 14, 30, 49, 71). This includes evaluating the prevalence of exhausted, metabolically dysfunctional, or epigenetically stabilized T-cell populations at baseline, as well as the presence of myeloid-dominant suppressive circuits. Importantly, these features may exist independently of tumor mutational burden or PD-L1 expression, which remain widely used but incomplete biomarkers for ICI response prediction (72–74). This organ-context view may also help explain why selected combination regimens, including anti-PD-1 combined with CD40 agonism, CSF1R blockade, myeloid-targeting approaches, or metabolic reprogramming, can improve antitumor activity in specific resistant or inflammatory settings (75–78). The success of these approaches may reflect their ability to reset or redirect immune rewiring trajectories, rather than merely deepen checkpoint blockade.

### Evidence hierarchy and unresolved questions

Although evidence supporting organ-context-dependent checkpoint responses is not equally strong across organs and tumor types, in MASH-associated HCC, preclinical and translational studies directly link metabolic liver inflammation, pathogenic CD8^+^ T-cell programs, and impaired checkpoint responsiveness (12). Evidence from colitis-associated colorectal cancer is more limited and largely preclinical, whereas COPD-associated NSCLC shows heterogeneous clinical associations, including reports of improved ICI outcomes in patients with mild or moderate COPD or CT-defined emphysema. Glioblastoma illustrates immune exclusion and checkpoint resistance rather than a classical HPD model.

While the most direct clinical evidence linking inflammatory or immunoregulatory markers to HPD comes from HCC, this evidence remains mostly associative (52, 79, 80). In advanced HCC treated with nivolumab, HPD was observed in 12.7% of patients and was associated with poor survival and elevated baseline neutrophil-to-lymphocyte ratio (52). Cross-tumor and HCC-focused meta-analyses further support an association between elevated neutrophil-to-lymphocyte ratio and HPD risk, although this marker reflects systemic inflammation and does not prove that the inflamed organ microenvironment directly causes HPD (79, 80). Mechanistic support comes from complementary models in which PD-1 blockade expands pathogenic CD8+ T-cell populations in MASH-associated HCC, amplifies PD-1+ regulatory T cells, permits rapid progression in Treg-dominant tumor microenvironments, or engages macrophage Fc/FcR-dependent pathways in NSCLC (10–12, 81). Together, these studies support the possibility that pre-existing inflammatory, myeloid-rich, or immunoregulatory immune states can bias post-ICI trajectories toward HPD-like acceleration in selected contexts, but they do not establish chronic inflammation as a universal cause of HPD.

These differences are central to the organ-context model: tissue immune state may condition checkpoint response, but it does not act alone or in a uniform direction. Prospective studies with pretreatment growth kinetics, serial imaging, baseline immune profiling, and post-treatment tissue or blood-based sampling will be needed to distinguish treatment-associated acceleration from aggressive natural history and to test whether specific tissue immune states predict HPD risk.

### Stratification and trial design

Current stratification strategies in immunotherapy trials rely on tumor-centric features, including histological subtype, genomic alterations, tumor mutational burden, components of antigen presentation machinery and markers such as PD-L1 expression (41, 82). However, even when tumors are classified as immune-inflamed or immune-excluded, these categories may not fully capture the immunological constraints imposed by the host organ (28). A shift toward organ-context-aware stratification would therefore not replace existing immune profiling, but extend it by integrating tumor immune state with the inflammatory, tolerogenic, metabolic, or myeloid architecture of the tissue in which the tumor arises (33, 45, 83). In practice, this would require prioritizing measurements that resolve immune cell spatial organization, T-cell functional state, myeloid composition, and systemic inflammatory tone, rather than relying on infiltration density alone. For example, in preclinical models of MASH-associated HCC or colitis-associated colorectal cancer, biomarkers of maladaptive CD8^+^ T-cell activation and myeloid skewing have been associated with paradoxical outcomes following PD-1 blockade that might argue against monotherapy ICI and support combinatorial strategies (12, 20, 49). This approach would also necessitate revisiting clinical endpoints and response kinetics (84, 85). In organs prone to maladaptive immune activation, early tumor progression following ICI may reflect true immune-driven acceleration rather than pseudoprogression or natural history, necessitating refined criteria for benefit assessment (2, 3). However, because HPD definitions vary across studies, future trials should predefine HPD criteria, include pretreatment growth-rate estimates when available, and separate primary progression, pseudoprogression, early treatment failure, and true acceleration of tumor growth.

### Conceptual reframing in immuno-oncology

Many immuno-oncology strategies aim to restore or enhance antitumor immune activity (23, 28). However, findings across the liver, colon, lung, and brain suggest that the therapeutic barrier is not always insufficient immune activation, but can also involve maladaptive immune organization (12, 20, 30, 32, 62). In several tumor-bearing tissues, immunity may be present but functionally misdirected, metabolically stressed, spatially constrained, or dominated by suppressive myeloid and regulatory circuits. Checkpoint release in such conditions risks amplifying immune circuits that are non-productive, even pro-tumorigenic, rather than restoring antitumor immunity (12, 20, 30, 32, 62).

This distinction shifts the central question from whether immunity can be activated to whether the existing immune state is recoverable, suppressive, maladaptive, or inaccessible. Chronic inflammation, immune tolerance programs, and metabolic or fibrotic dysregulation can create immune landscapes that are already active but maladaptive, such that checkpoint release may favor pathological rather than protective immune activity. At the same time, the lung and colorectal cancer literature show that inflammation is not intrinsically detrimental: specific immune states can coincide with checkpoint responsiveness when antigenicity, T-cell infiltration, spatial organization, and recoverable effector function are present.

These observations argue for biomarkers and stratification strategies that assess immune quality, not only immune quantity. Tumor-centric biomarkers remain important, but they may be insufficient when used without information on immune contexture, cellular programs of exhaustion or dysfunction, myeloid composition, spatial organization, systemic inflammatory tone, and tissue-specific tolerogenic constraints. In this view, successful immunotherapy may require identifying the immune state in which checkpoint release occurs, then pairing checkpoint blockade with interventions that redirect maladaptive immune activity toward productive antitumor immunity.

## Data Availability

The original contributions presented in the study are included in the article/supplementary material. Further inquiries can be directed to the corresponding author.
